# Medical Men as Prisoners of War

**Published:** 1914-11-07

**Authors:** 


### The Hospital

The Workers' Newspaper of Administrative Medicine and Institutional Life, Administration, National Insurance and Health.

No. 1481, Vol. LVII. SATURDAY, NOVEMBER 7, 1914. PRICE ONE PENNY.

### MEDICAL MEN AS PRISONERS OF WAR.

The present war, in harmony with previous experience, has shown that the medical men in the fighting line, though technically distinguished as non-combatants, are liable to all the dangers and hardships which attend the services of the fighting ranks. No one will wish to make comparisons between men who share a common danger for the sake of a great and unselfish cause. At the same time, it is fair to remark that the medical officer cannot have, as a helpful and sustaining force, the full glow and stimulus of combatant effort. He must take his chance of blows without the sense of satisfaction which comes from an effort to pay back his assailants in kind. Alike amid the *élan* of victory and the gloom of defeat his duties remain unaffected, and to these he must devote himself whatever be the immediate military atmosphere and environment. All this makes a great demand on the steady heart and the power of self-control. It needs what has been called "two-o'clock-in-the-morning" courage—a courage which owes little or nothing to external circumstances or to the sense of support by others who share a common enterprise. The country generally, and our medical readers least of all, are not likely to forget the quiet and steady heroism which secures, so far as this is possible, prompt succour for those who fall wounded on the field.

That the risks to which we here refer are real is made painfully evident by the officially authorised casualty lists. Medical officers not a few have met death in the firing line, and in paying a word of tribute to their memories we would equally recall the less conspicuous, but not less heroic, members of their corps, who, while acting as "bearers," suffer all the chances of the field of battle. They, also, though without arms in their hands, have fought a good fight and have deserved well of their country.

One of the chances to which medical, equally with combatant officers, are exposed is the risk of being taken prisoner. Indeed, the medical officer's liability in this respect is peculiarly great, seeing that his movements cannot be governed purely by military considerations. His place is primarily not where the army is moving, but where there are wounded men. The influence of this factor becomes specially prominent when the army with which the officer is serving is in retreat. Necessarily in such circumstances attention to the wounded means exposure to capture by the advancing enemy, and in this fashion is explained the number of officers of our R.A.M.C. who were taken prisoners during the early weeks of the war. It is this consideration which we make the first ground of an appeal that medical officers engaged in the succour and care of wounded soldiers, and taken prisoners when so occupied, should be released at the earliest possible moment. The misfortune which has overtaken them is a consequence of the degree of devotion with which they have pursued duties dictated not so much by military considerations as by the claims of humanity. Personal safety, and even the wishes of military chiefs, suggest that the medical corps shall retreat with the other parts of the army. Yet in individual instances obligations of a supreme order make this impossible, and the medical officer yielding to these becomes a ready prey to the advancing enemy. Surely the faithful discharge of a duty which is non-combatant in character and which is in no sense directed against the enemy's advance ought not to entail, when these can be avoided, the penalties and misfortunes of war? The bullet the shell, and even the bayonet and the lance—with these the army doctor must take his chance; but it is not necessary to add to these the weariness and hardships which attend more or less those who are prisoners of war.

There is another reason which may be urged in favour of releasing medical prisoners of war—a reason, we mean, which may be fairly expected to appeal to the military mind. It is that the medical officer is ready to give, and as a matter of fact does give, his professional help and services to the wounded of all the combatants. His engagement, except in the narrowest technical sense, is not to the flag or the uniform, but to those, whoever they be, who are in need of his help. Hence to diminish the number of medical officers in the field, as by holding some proportion of them prisoners of war, is to limit the amount of help, not to one combatant only, but to all the combatants. Even on the ground of patriotic self-interest, therefore, there is reason for the release of all medical officers who in the changes and chances of the struggle become prisoners of war.

Last, but by no means least, is the supreme motive which makes its appeal to humanity in the figure of the Good Samaritan. Even amid all the horrors of modern war there is surely abundant evidence that this appeal has lost none of its native and original force. And yet, in spite of it—such is the mental confusion of the position—medical officers, because technically they are soldiers, are rendered idle and useless by an enforced captivity, while on the battlefield and in hospital sick and wounded men are suffering and dying for lack of help. Can any contrast be more pathetic or any situation more bitter? Everybody is willing to allow that in the stress of war military necessities must be pressed even at the expense of personal sufferings. But in the instance now before us there are no military considerations except in a remote and secondary sense. We are sure that no soldier would say that his wounded enemy ought, if possible, to be deprived of medical aid. Yet this, in substance, is what the present practice affirms. Let us have done as soon as possible with a bad tradition, and be ready to honour the nation who will first set a good example by restoring promptly to their beneficent duties all medical officers who are made prisoners of war.

